# The experience of living with chronic heart failure: a narrative review of qualitative studies

**DOI:** 10.1186/1472-6963-10-77

**Published:** 2010-03-24

**Authors:** Yun-Hee Jeon, Stefan G Kraus, Tanisha Jowsey, Nicholas J Glasgow

**Affiliations:** 1 The Australian Primary Health Care Research Institute; Menzies Centre for Health Policy, The Australian National University, Building 62, Mills Rd, Canberra, ACT 0200 Australia; 2 Sydney Nursing School, The University of Sydney, Sydney, 88 Mallett Street, Camperdown NSW 2050 Australia; 3 Medical School, The College of Medicine, Biology and Environment, The Australian National University, Frank Fenner Building 42, Canberra, ACT 0200 Australia

## Abstract

**Background:**

Chronic heart failure (CHF) is the leading cause of all hospitalisations and readmissions in older people, accounting for a large proportion of developed countries' national health care expenditure. CHF can severely affect people's quality of life by reducing their independence and ability to undertake certain activities of daily living, as well as affecting their psychosocial and economic capacity. This paper reports the findings of a systematic narrative review of qualitative studies concerning people's experience of living with CHF, aiming to develop a wide-ranging understanding of what is known about the patient experience.

**Methods:**

We searched eight relevant electronic databases using the terms based on the diagnosis of 'chronic heart failure', 'heart failure' and 'congestive heart failure' and qualitative methods, with restrictions to the years 1990-May 2008. We also used snowballing, hand searching and the expert knowledge of the research team to ensure all relevant papers were included in the review. Of 65 papers collected less than half (n = 30) were found relevant for this review. These papers were subsequently summarised and entered into QSR NVivo7 for data management and analysis.

**Results:**

The review has identified the most prominent impacts of CHF on a person's everyday life including social isolation, living in fear and losing a sense of control. It has also identified common strategies through which patients with CHF manage their illness such as sharing experiences and burdens with others and being flexible to changing circumstances.

Finally, there are multiple factors that commonly impact on patients' self care and self-management in the disease trajectory including knowledge, understanding and health service encounters. These health service encounters encompass access, continuity and quality of care, co-morbid conditions, and personal relationships.

**Conclusions:**

The core and sub-concepts identified within this study provide health professionals, service providers, policy makers and educators with broad insights into common elements of people's experiences of CHF and potential options for improving their health and wellbeing. Future studies should focus on building a comprehensive picture of CHF through examination of differences between genders, and differences within age groups, socioeconomic groups and cultural groups.

## Background

In 2005 approximately 30% of global deaths (17.5 million) were attributable to cardiovascular disease (CVD) [1]. Chronic heart failure (CHF) significantly contributes to this disease burden and is the leading cause of all hospitalisations and readmissions in older people, accounting for a large proportion of developed countries' national health care expenditure [2,3]. The estimated prevalence of CHF in people aged 45 years or more ranges between 3 and 5% worldwide [2], although the true prevalence of CHF may be higher due to under-diagnosis of mild to moderate CHF. The majority of CHF patients are females, largely attributable to their longer life expectancy. Higher rates of CHF are also reported in older people, with a tripling of the rate for those 75 years or over compared with the rate in the 55-64 years age group [2,4].

CHF occurs when the heart's blood pumping function is compromised resulting in under-perfusion of tissues. It may result from a number of underlying conditions including ischemic heart disease, hypertension, valvular heart disease, cardiomyopathies, and congenital heart disease. CHF often coexists with diabetes and kidney related disease [5]. CHF leads to progressive physical and functional deterioration resulting in shortness of breath, tiredness, weight gain due to fluid build-up, swelling of ankles or abdomen, dizziness, and intermittently unpredictable life-threatening crises, requiring repeated hospitalisation.

There is no cure for CHF. In its most severe form CHF has over 50% one-year mortality [6]. Management targets symptoms and lifestyle modifications. It can severely affect people's quality of life by reducing their independence and ability to undertake certain activities of daily living, as well as affecting psychosocial and economic capacity.

Therapeutic advances in medical and pharmacological interventions for heart failure, together with changes in patterns of health care practice, have transformed the experience of many patients with CHF [7]. However, the economic and social burden of disease on the community remains high [4], and the multifaceted impact of CHF continues to overwhelm those individuals and families affected by the disease [8,9]. Chronic care management programs such as care coordination and care/case management often target people with CHF, diabetes, asthma, chronic obstructive pulmonary disease (COPD), and/or hypertension to provide more integrated and continuous care [3]. Despite variations in structure, scope, delivery and location (hospital-based or community based), the primary aim of chronic care programs is to improve the health and well-being of people affected by chronic illness and decrease avoidable hospital admissions [3]. The outcomes of such programs tend to focus on the pattern and the level of service utilisation and associated costs to the governments or health system, often by examining reductions or increases in avoidable emergency presentations and hospitalisations/readmissions and length of hospitalisation [3,10-13].

Patient-centred care is a central idea in health system reform. Patient-centred care helps patients and carers actively participate in care and clinical decision making, based not only on their disease state but also on uniqueness of culture, language, preferences, mores, values and attitudes [14]. In their examination of 129 systematic reviews of patient-centred interventions, Coulter and Ellins [15,16] conclude well-designed measures that actively engage patients in their health care decision making can facilitate improved health literacy, experiences with care resources, better health behaviours and improved health. However, the best way of embedding patient-centredness in policy and practice remains the subject of debate.

The Serious and Continuing Illness Policy and Practice Study (SCIPPS) was designed to develop patient-centred policy and health systems interventions and provide optimal patient care and carer support in chronic illness. SCIPPS focuses on a population aged 45-85 years old, with one of three chronic conditions--diabetes, CHF and COPD. Conducted as an initial part of SCIPPS, a preliminary literature search on the experience of people with chronic disease(s) showed common threads of such experience across countries, ages, gender and cultures. This paper reports on the findings of a systematic narrative review of qualitative studies concerning people's experience of living with CHF. The aim of this review was to develop a wide-ranging understanding of what is known about the patient experience of CHF based on narrative accounts of firsthand experience by patients or indirect experience by health professionals or family carers.

## Methods

We defined qualitative studies as those aiming to uncover and understand a phenomenon, a process, or perspectives and worldviews of people, with or without a particular theoretical orientation, using typical qualitative methods for sampling, collecting, analysing, and interpreting data [17]. In systematic steps of search and collating information, we chose an inclusive approach to the process, reflecting the diversity of qualitative studies based on topic relevance, degree of methodology confidence and depth of information, including papers providing particular insights. We noted a similar review of fourteen qualitative studies on the older person's experience of chronic heart failure by Yu et al. [9] providing an interpretation using the transactional model of stress. Unlike Yu et al. [9] given the diversity of the population with CHF and the multifaceted experience these people have, we felt it was important to include studies using various adult populations without specifying a particular theoretical framework.

### Search methods

We searched eight relevant databases using the terms in Table 1 with restrictions to the years 1990-May 2008. Search terms used in each database were based on the diagnosis of chronic heart failure and qualitative methods. Distinctions between the terms heart failure, chronic heart failure and congestive heart failure are at times unclear and often used interchangeably in the literature. Given this problem we included heart failure, chronic heart failure and congestive heart failure. We ensured those search terms were found in one of the four domains of the electronic search systems to enhance sensitivity and specificity including: ab. (word in Abstract), hw. (word in the Subject heading word), kw. (word in Keyword), and ti. (word in Title). We also used snowballing, hand searching and the expert knowledge of the research team to maximise search results.

**Table 1:** List of databases and search terms utilised

DATABASES (Between 1990 May 2008)	LIST OF SEARCH TERMS (ab., ti., kw., hw.)	SELECTION CRITERIA
CINAHL MEDLINE PsycINFO The Cochrane Central Register of Controlled Trials APAIS Health Proquest Health Wiley Interscience PubMed	'Chronic heart failure' or 'congestive heart failure' or 'heart failure' AND 'experience' AND 'Qualitative', 'Interview', 'Phenomenology', 'Grounded Theory', 'Narrative', 'Ethnography' or 'Observation'	Inclusion Criteria: Literature describing the experiences of people diagnosed with CHF: 1) people with CHF, 2) carers of people with CHF, and/or 3) health care professionals involved in caring for patients with CHF
		
		Exclusion criteria: Editorials, commentaries, discussion papers and conference abstracts Non-English paper

### Selection process

There have been numerous attempts to establish definitive and objective criteria for quality appraisal of qualitative studies and standard synthesis [18,19]. Nevertheless, challenges still remain when ranking and selecting qualitative studies based on their methodological rigour due to extreme heterogeneity of methodological descriptions and editorial requirements of published qualitative papers. This is further complicated by the intricacy of achieving consensus about highly subjective matters that require methodological and practical experts--and they may have differing worldviews or disciplinary backgrounds. Therefore, we chose to include a diverse set of qualitative studies encompassing various descriptions and interpretations of the CHF experience.

### Data abstraction and synthesis

An initial literature search (n = 57) and a process of snowballing (n = 8) delivered a set of 65 references. Of these, 42 were reviewed by the first author (Y-HJ) using titles and abstracts and were identified as relevant using inclusion/exclusion criteria. Another research team member (SK) then retrieved and read the full-text articles to confirm the relevance of each, deeming 12 more to be irrelevant (confirmed by Y-HJ). The remaining 30 articles were summarised by SK using a template noting referencing information, study setting, sample characteristics, study design and methods, key findings, barriers/facilitators to the management of CHF and recommendations. The summaries were then imported into QSR NVivo7, a useful data management tool for large scale text information [20]. The summaries of the final 30 papers were reviewed by YHJ, and a coding scheme comprising 70 thematic codes was compiled with definitions for each concept. Each article was then coded with this scheme by SK.

Next, all 30 articles were read again by Y-HJ, SK and TJ, and the thematic codes were discussed and rearranged. During this process it became clear that the experiences of people with CHF found in those 30 articles could be encapsulated under the following three questions, which appropriately encompassed all of the thematic codes:

1. What can be deduced about the common impacts of CHF on a person's everyday life?;

2. What can be deduced about the common patterns of coping strategies people living with CHF develop and utilise?; and

3. What are the factors influencing self care and disease management?

We then critically examined the thematic codes against these three key questions, resulting in 10 core concepts and 51 sub-concepts. The sub-concepts contributed to the explication of the 10 core concepts, which ultimately shaped our findings. Table 2 shows the final list of core and sub-concepts.

**Table 2:** Core concepts and Sub-concepts

Central Concepts	No of papers (n = 30)	Sub-concepts
**1. Impact of CHF on everyday life**		

Social isolation	20	Feeling abandoned; Physical restrictions; Food and diet; Medication; Fatigue; Relationships with family and friends

Living in fear	16	Uncertainty; Frustrated; Sleep; Work restrictions; Being limited; Behaviour change

Losing a sense of control	15	Symptoms; Being limited; Helplessness; Unpredictable

**2. Common patterns of coping strategies**		

Sharing experiences	13	Practical support; Psychological support; Emotional support; Knowledge; Assistance; Friends/family; Comfort

Being flexible to changing circumstances	14	Coping; Adjustment; Awareness; Acceptance; Making changes

**3. Factors influencing self care and/or the provision of good care**		

Knowledge	17	Knowledge; lack of knowledge; Acquiring information; Self-management; Self care; Emotional benefit; Navigating health services; Access to services

Health Services -- availability and access	3	Time constraints; Communication; Negative experience; Patient satisfaction

Health Services -- continuity and quality of care	15	Advice; Self care; Time constraints; Self care; Trust; Multiple care providers; Conflicting advice; Confusion; Education

Co-morbidity	11	Depression; Diabetes; Arthritis; Dietary restriction; Exercise; Sexual life

Personal Relationships	26	Family; Friends; Sexual life; Peers with CHF; Changing roles; Social isolation

## Results

Of the 30 studies included, 27 examined the experiences of people with CHF in a general context and three examined it in the context of intervention studies [21-23]. As shown in Additional file 1, the majority of selected studies were conducted in the United States (n = 14) and Sweden (n = 11) and represented a variety of settings including hospital (n = 6), community (n = 7) and outpatient clinics (OPC), alone or combined with other settings, (n = 15). Most focused on patient perception only (n = 25), as opposed to the perspectives of health professionals or family carers. Eight studies used a phenomenological approach, three used phenomenography, five used grounded theory, thirteen used a generic qualitative design and one used an ethnographic approach. The size of the samples ranged from 1 to 87.

Some studies had a specific focus in the context of the experience of CHF, including: adherence to self care/self-management [24-28]; quality of life and spirituality [29,30]; specific types of care or care settings such as palliative care [31], nurse led clinic [22], nursing home [32] and hospital care [33]; gender differences or gender specific [32,34-40]; cultural diversity or specific ethnicity [23,24,41]; health seeking behaviour [39]; and fatigue [36,42].

### 1. Impact of CHF on everyday life

The 30 studies revealed various experiences common to patients with CHF that impacted on everyday life with the most prominent being social isolation, fear and loss of control.

#### Social isolation

Twenty studies reported social isolation as a key concept. It could arise from various factors including lifestyle changes, medication regimens, fear, and physical restrictions due to shortness of breath and fatigue. In Bentley et al.'s study [25], participants reported inability or limited capacity to participate in social events, limited opportunities to socialise with friends and family, and being misunderstood and disrespected because of dietary restrictions. Struggling with isolation was an ongoing problem for patients, not only due to physical limitations but due to feeling abandoned or let down by family and friends [31].

Undesired or adverse pharmacological effects also contributed to social isolation. The use of diuretics to address excess body fluid, for example, produced urgency and frequency of urination leading to sleep deprivation and preventing people from leaving home and/or socialising [24,43]. Fatigue, often accompanied by weakness and unpredictable variations in physical ability, sometimes caused by medication, had similar implications for social isolation by reducing patients' abilities to participate in recreation and leisure [35,36,42,44]. Travelling, for example, proved too strenuous for many people with CHF and resulted in patients feeling like prisoners in their own homes [42].

#### Living in fear

Living in fear of pain, death or the future was reported as a central aspect of patient CHF experience in sixteen studies. These studies discussed fear in terms of 'affective response' [30], 'visiting death's door' [31] 'emotional burden' [35], 'to live with thoughts - past, present and future' [42], 'feeling anxiety' [38], 'distressed emotions' [26], 'patient's response to the diagnosis' [45] and 'emotional turbulence' [43]. Studies reported patients limiting physical and social activities due to fearing personal safety and inability to manage or control situations [24,44,46]. Females and males voiced fears with different emphases. Females reported fear more frequently than males [24], often describing being worried and fearful about dying while asleep, which kept them awake at night [24,44]. In a study of 20 men [34], participants reported fear of inability to continue work as their first concern upon diagnosis, however as the disease progressed they became more fearful of death, particularly during episodes of dyspnoea. A study comparing gender differences in experience of CHF reports that men tended to be negative and pessimistic towards their illness and situations, while women were likely to be accepting and optimistic [35]. However, findings from other studies suggest such differences may not necessarily be gender specific [30,37-39].

#### Losing a sense of control

Sense of control was found to be an important factor in the lives of patients with CHF. A sense of control occurs when patients perceive they have the power and capacity to influence their life or their illness. Patients who felt they had a sense of control through taking responsibility for lifestyle changes were reported as connecting this sense of control with feelings of satisfaction and inspiration [26], or for some patients it was a sense of transcending the illness [47]. Patients losing a sense of control over their illness were reported as connecting the loss of control with unpredictable deterioration in health, high blood pressure, shortness of breath and sleeplessness; and over their life in terms of loss of independence, financial security and participation in CHF management decision-making [22,31,36,40,44]. Losing this sense of control, or 'feeling imprisoned in illness', as Ekman described [47], was also associated with various restrictions imposed on their lives due to the need to adhere to disease management, resulting in feelings of helplessness, powerlessness and that premature death was unavoidable [21,36,37]. A phenomenological study found that the person's experience of losing a sense of control and self confidence was exacerbated by relocation, such as from home to a nursing home, and the way in which care was given and independence diminished in the new care setting [32].

### 2. Common patterns of coping strategies

In response to the emotional, psychosocial and physical impact of illness patients develop diverse coping strategies to alleviate the impact on their lives. The two most common coping strategies identified were sharing the illness experience with others and being flexible to changing circumstances. Others less commonly discussed included remaining hopeful or inspired religious faith, undertaking positive self-talk and denial.

#### Sharing experiences (and burdens with others)

Sharing experiences and burdens with others involves people accepting actions and activities by others that ease the multiple challenges and provide support in practical, psychological or emotional ways. Thirteen studies identified sharing the illness experience as a significant coping strategy for people with CHF. Sharing experiences was explored in terms of getting social support, working in collaboration or working things out with others, getting practical support (e.g. assistance with transportation and shopping) [28,40,46,48]. Sharing experiences was perceived as a necessity that patients sometimes resented because it represented a loss of independence, control and role identity [21,30]. However, sharing experiences of CHF with spouses and other family often brought them closer, strengthening relationships [30,48]. Sharing experiences with peers was noted in several studies as an important source of support; providing an opportunity to exchange information with people who had 'been through it' [21,28]. Support groups in particular provided a sense of validation, relief, comfort and camaraderie, as well as a means of overcoming social isolation through meaningful social activity [21,30,41,46].

#### Being flexible to changing circumstances

CHF requires significant changes and several studies noted that patients frequently encountered difficulty coming to terms and making necessary adjustments. Being flexible to changing circumstances requires that patients be aware of and accept physical limitations and new life situations; and adapt or adjust activities to make the most of the situation. For example, patients talked about completing activities at a slower pace, changing hobbies/physical activities requiring less effort, staying quiet, dimming lights, resting between activities or finding less energy-demanding ways to complete tasks [24,30,37].

### 3. Factors influencing self care and/or the provision of good care

Common factors contributing to patients' self care capacity and the quality of received care includes knowledge and understanding, health service encounters including access, continuity and quality of care, co-morbid conditions, and personal relationships.

#### Knowledge of CHF and its management

Seventeen studies discussed knowledge associated with self care or provision of good care for people with CHF as a key factor in successful management of CHF. Fourteen of these studies collectively reported that 'insufficient knowledge' or 'lack of understanding' resulted in poor care outcomes, unrealistic patient expectations and confusion over what to expect and how to manage changes in their condition, eliciting anxiety and frustration. Insufficient patient knowledge of their illness and effective management also led to non-adherence of self-management strategies, such as exercise or low-sodium diet [25], inappropriate self care [26], and delays in seeking care [23]. Beutow et al. [49] found that some patients actively avoided acquiring information as a strategy for 'emotion-focused coping' rather than 'disease-focused'; hence they did not understand the nature or seriousness of their illness. One Australian study examining cultural issues of seven ethnic groups of people born overseas reported particular challenges due to lack of knowledge concerning health services. Patients and families in this study expressed the importance of the family doctor's role and family involvement, as well as support in navigating health services including the referral and payment systems and health-related decision making [41].

Increasing patient knowledge about self care was reported as having a positive influence over patient health and well-being [21,23,26,28]. In a behavioural intervention study, patients receiving CHF education on techniques to elicit a relaxation response experienced physical and emotional benefits [21]. Similarly, knowledge and learning about CHF facilitated care, specifically in terms of patients' ability to connect their action with outcome, medication side-effects, and interpret symptoms to seek appropriate care [23,26,28].

#### Health service encounters - availability, access, continuity and quality

Difficulties with accessing health care services were reported across all care settings. One third of the studies reported patients experiencing limited availability and access to services, often due to time constraints on health care providers and inefficient referral arrangements between physicians and specialists. Patients reported their physicians were often hard to contact, rushed or uninformative, inattentive to their needs and problems, and referrals seemed to take unnecessary time to process [26,31,50].

Half the studies in this review (n = 15) provided key findings associated with the continuity and quality of care experienced by patients. Examples of good care practice included: showing concern and providing consistent advice to both patients and family members; offering practical strategies for self care; being knowledgeable about diagnosis and management procedures; and allowing consistent access to the same care provider over time (e.g. [25,28,38,50]). When patients experienced good quality of care they developed confidence and rapport with care providers, found care providers easy to approach, and felt safe and secure (e.g., [27,28,31,39,46]). The positive involvement, concern and advice of healthcare professionals helped patients seek timely care and maintain self care practices.

Studies also reported poor and inappropriate care practice, in terms of health professionals not engaging patients in their care and decision making, patients not receiving sufficient information about diagnosis or condition management, insensitive approaches to female patient needs, and improper medication scheduling (e.g., [25,26]). Other examples of poor care practice included health providers creating unnecessary fears, not tending to immediate needs such as toileting assistance and ignoring patients (e.g. [26,33]). When patients experienced poor quality of care they reported lack of confidence in care providers, confusion, delays in seeking care and were deterred from maintaining positive self care practices. Naturally, prior negative experience of accessing services discouraged patients from seeking timely help [31].

#### Co-morbidity

Co-morbidity refers here to the presence of more than one illness impacting on the management of CHF and the patient's life. Eleven studies listed co-morbid conditions in their key findings. The most commonly reported co-morbid conditions impacting on patient ability to manage CHF were depression (n = 9), diabetes (n = 5), arthritis (n = 3) and asthma (n = 2). Co-morbidity influenced CHF management by masking CHF's early stages, causing late diagnosis [50] and complicating patients' attempts to manage their illness, in particular lifestyle changes and diet [40,48]; and increasing risks of undesired pharmacological interactions and adverse effects of medications, ACE inhibitors and beta-blockers [40].

Depression was reported to decrease patient hope, self worth and frequency of socialisation, increasing feelings of helplessness and isolation [21,30]. Diabetes was a common co-morbid condition in people with CHF, one study reporting approximately one third of participants (n = 26) had diabetes [26]. When patients had CHF and diabetes they found it difficult to follow dietary restrictions for both CHF *and *diabetes [25]. Arthritis restricted patients' ability to manage CHF through exercise [21]. Asthma was also connected with possible drug-related contraindications [50] and patients encountered difficulty discerning between exacerbations of asthma and exacerbations of CHF [48].

#### Personal relationships

Twenty-six articles identified changes in family roles and personal relationships due to unavoidable increase in patient dependency and changing needs (e.g. [24-26,28,30,36]). Collectively the studies reported negative patient emotional responses to changes in roles and relationships, such as guilt, anxiety, frustration, uncertainty, fear, loneliness and feeling useless. Patients felt guilt and anxiety over being a burden on others, dependency caused frustration and uncertainty as well as fear of inadequate support provision (e.g. [30,35,38]). Some patients experienced being misunderstood by friends and family who did not appreciate the severity of their condition and this was interpreted as a potentially negative effect on patient quality of life [30]. CHF affected sexual performance, frequency and intimacy [24,30,43]. Bennett et al. [24] found that male participants frequently reported "loss of interest in sexual activities or sexual performance problems" while female participants reported no relationship between CHF and sexual activities.

Studies also reported positive feelings of safety and reliability associated with changes in personal relationships, especially where the partners shared responsibility for managing the illness, such as helping patients adhere to dietary requirements or accompanying them to health services [36,46].

## Discussion and Conclusions

Despite the diversity in characteristics of study participants' cultural background, age and gender, care settings, and the country where the study was conducted, people affected by CHF share similar experiences of emotional, psychological, social, relational and physical consequences. This review has identified common strategies through which patients with CHF manage their illness and multiple factors (families/friends, health services/health professionals and co-morbidities) that commonly impact on their self care disease management in the disease trajectory.

The core and sub-concepts identified within this study provide health professionals, service providers, policy makers and educators with broad insights into common elements of people's experiences of CHF and potential options for improving their health and wellbeing. It thus has the potential to enhance implementation of patient-centred care. Improving peoples' knowledge of their condition including medical and health service aspects are all important in self care and self-management interventions. The psychosocial aspects of care (i.e. social isolation, fear, sense of identity and control, depression, and personal relationships) must also be addressed to optimise care for people with CHF.

Most studies were heterogeneous in their sample characteristics, some not even providing information on participants. Please refer to Additional file 1 for details of the studies included in this review. Whilst studies of certain age groups, gender or ethnicity/race provide more specific information about the experience of a particular group, their utility in a broader context is limited because of their lack of systematic approach or consistency in comparing the groups within the study. Future studies should focus on building a comprehensive picture of CHF through examination of differences between genders, and differences within age groups, socioeconomic groups and cultural groups.

A good qualitative study entails the power of describing, explaining and theorising the phenomenon of interest. More focus should therefore be placed on a theory development of the process of living with CHF that explains the complex relationships between and within the key concepts associated with individual characteristics (e.g., age, gender, and culture).

We limited this review to qualitative studies and the quality or rigour of the papers was not specifically assessed for the reasons described earlier. This open approach, however, has allowed us to identify and confirm the commonly occurring concepts across various settings and sample characteristics based on a larger pool of studies.

We acknowledge that we inadvertently excluded several relevant qualitative studies due largely to electronic system errors and partly to our search strategy, which restricted findings by requiring the inclusion of the word 'experience'. We discovered this following the completion of the review by comparing our search outcomes with those of a recently published review on a similar topic by Welstand [51]. Examination of the concepts described in those additional references included in Welstand et al.'s review is consistent with the core and sub-concepts identified in our review, confirming the validity and relevance of our findings. In addition, when compared with Welstand et al.'s search strategy, which used a broad range of search terms, our method of combining a more targeted search strategy with additional hand searching and snowballing methods identified a larger pool of studies for review.

Given the similarities in subject matter and methodology chosen by the current study and those chosen by Yu et al. [9] and by Welstand et al. [51], distinctions between these studies must be made. Using an integrative or inclusive review approach Welstand and her colleagues [51] unpacked the experience of CHF under five concepts, namely: 'Diagnosis and manifestations of heart failure', 'Perceptions of day-to-day life', 'Coping behaviours', 'Role of others', and 'Concept of self', which is identified as an overarching concept influencing the other four. Yu et al. [9], on the other hand, attempted an interpretation of the experience of older people with CHF using the transactional model of stress, which they summarised under three headings of 'Perceptions of the manifestation of CHF', 'Coping strategies when living with CHF', and 'Adjustments to living with CHF'.

On the surface, the key headings/conceptual categories of these studies may appear quite similar to those of the current study due to overlaps in the areas of 'Impact of CHF on everyday life' and 'Coping strategies' identified in our review. The earlier two studies attempted to develop some form of theoretical interpretation of the phenomenon, or discovery of a particular process that people with CHF undergo, either by using a predetermined theory of stress and coping as an analytical lens [9], or by focusing on elaboration of emerging conceptual categories and linking them to a particular process of people with CHF taking on a new identity [51]. The current study, however, has attempted to provide a wide-ranging, descriptive, and nevertheless practical, overview of what has been reported in the existing qualitative literature on the experience of people with CHF. In doing so, and in quantifying the trends of qualitative research on the subject matter and summarising the findings under the key questions, we have elicited the most commonly reported factors that influence self care and the provision of good care, as well as the impact of CHF and coping strategies. Critique of the two earlier reviews is beyond the scope of this paper, however we are confident that our review has provided additional insights to the existing knowledge as well as some key answers that are often overlooked in the process of theorising or conceptualising the human experience.

## Competing interests

The funding organisation (NHMRC) had no role in the study design, data collection, analysis and interpretation, or the writing and publication of this article. The authors declare that they have no competing interests.

## Authors' contributions

Y-HJ conceived and designed the study, participated in all stages of data extraction and synthesis, and provided supervision of SK and TJ. SK conducted an initial literature scoping exercise and synthesis together, collated and summarised articles under close supervision of Y-HJ. Y-HJ, SK and TJ drafted different sections of this paper, and Y-HJ revised the paper as a whole and contributed to revisions and the final version of the manuscript. NJG contributed to the early design concepts for the narrative review and actively contributed to the writing and production of the final manuscript. All authors contributed revising various versions of the manuscript. All authors read and approved the manuscript.

## Pre-publication history

The pre-publication history for this paper can be accessed here:

http://www.biomedcentral.com/1472-6963/10/77/prepub

## Supplementary Material

Additional file 1 **A summary of qualitative research papers included in this review**. The table provides key attributes of each qualitative research paper included in this review. Click here for file
