# Supplementary material for: The experience of living with chronic heart failure: a narrative review of qualitative studies
Source: BMC Health Serv Res. 2010 Mar 24;10:77. doi: 10.1186/1472-6963-10-77 (PMC2851714; doi:10.1186/1472-6963-10-77)
Supplement: Additional file 1 — A summary of qualitative research papers included in this review. The table provides key attributes of each qualitative research paper included in this review. [file 1472-6963-10-77-S1.DOC]

## Additional File 1

Title: A summary of qualitative research papers included in this review

Description: The table provides key attributes of each qualitative research paper included in this review

| **Author**  **(Year)**  **Country (Setting)** | **Design/Method** | **Sample characteristics (Number of men)** | **Patient sample age group**  **(Years, yrs)** | **Diagnosis** |
| --- | --- | --- | --- | --- |
| Davidson et al. (2007)  Australia (Community) | Descriptive  (interviews and  Focus groups) | Patient (PT) & Family carer (FC) n=13 groups  (Gender not available)  Health care professionals (HCP) n=8 Other n=16 | Not available | Heart failure |
| Phillips et al. (2004)  Australia (General Practice) | Qualitative generic  (focus groups and telephone interviews) | HCP n=35 | Not applicable | Heart Failure  echocardiogram |
| Costello et al. (2004)  Canada (Hospital) | Qualitative generic  (interviews) | PT n=6 (3 men) | 33-73 yrs | Congestive heart failure  NYHA* functional stage III or IV |
| Simpson et al. (2000)  Canada (OPCπ and General Practice) | Qualitative generic  (focus groups) | PT n=26 (14 men) | Mean=66 SD=12 | Congestive heart failure. NYHA* functional stage I-III |
| Buetow et al. (2001)  New Zealand (General Practice) | Qualitative generic  (interviews) | PT n=62 (Not available) | Not available | Chronic heart failure  Treated for recorded heart failure for at least 2 months |
| Brannstrom et al. (2006)  Sweden (Palliative advanced home care) | Phenomenology  (interviews) | PT n=4 (3 men) | 72-81 yrs Median=79 | Chronic heart failure  NYHA* functional stage III or IV |
| Ekman et al. (1999)  Sweden (Hospital) | Phenomenology  (interviews) | PT n=12 (7 men) | 76-94 yrs Median=83 | Chronic heart failure  NYHA* functional stage III or IV and hospitalised for CHF on at least two occasions |
| Ekman et al. (2000)  Sweden (OPC) | Phenomenology  (interviews) | PT n=10 (6 men) | 75-94 yrs | Chronic heart failure  NYHA* functional stage III or IV |
| Ekman (2001)  Sweden (Nursing home) | Phenomenology  (interviews) | PT n=1 (Woman) | 79 yrs | Severe heart failure. |
| ^#^Eldh et al. (2006)  Sweden (Nurse-led OPD) | Phenomenology  (interviews) | PT n=3 (All men)  (all from the intervention group) HCP n=2 | PT=53, 77, 79 yrs | Chronic heart failure  NYHA* functional stage II or III |
| Europe et al. (2004)  Sweden (Community) | Qualitative generic (interviews) | PT n=20 (All men) | 43-73 yrs  Mean=59 SD=9 | Chronic heart failure  NYHA* stage II or III for more than three months, echocardiography  ejection fraction <0.40 |
| Falk et al. (2007a)  Sweden (OPC) | Grounded theory  (interviews and internet focus groups) | PT n=15 (8 men) | 31-95 yrs Mean=76 | Chronic heart failure  Aetiology of heart failure recorded. Severity not assessed. |
| Falk et al. (2007b)  Sweden (OPC) | Phenomenography (interviews) | PT n=17 (12 men) | 55-83 yrs Mean=72 | Chronic heart failure  NYHA* functional stage III or IV, hospitalised for CHF on at least two occasions |
| Hagglund et al. (2008)  Sweden (OPD) | Qualitative generic  (interviews) | PT n=10 (All women) | 75-89 yrs Mean=83 | Chronic heart failure  NYHA* functional stage III or IV, |
| Martensson et al. (1997)  Sweden (Hospital) | Phenomenography (interviews) | PT n=12 (All men) | 48-80 yrs | Congestive heart failure.  NYHA* functional stage II -IV |
| Martensson et al. (1998)  Sweden (Community and hospital) | Phenomenography (interviews) | PT n=12 (All women) | 65-83 yrs | Congestive heart failure  NYHA* functional stage II -IV |
| Bennett et al. (2000)  USA (OPC) | Qualitative generic  (focus groups) | PT n= 23 (16 men)  FC n=18 (17 women) | Mean=60 | Chronic heart failure by radiologic or echocardiography examination |
| Bentley et al. (2005)  USA(Community) | Qualitative generic  (interviews) | PT n=20 (12 men) | Mean=60.2 SD=10.7 | Heart failure due to left ventricular systolic dysfunction with ejection fraction <0.50 |
| Bosworth et al. (2004)  USA (OPC) | Grounded theory  (focus groups) | PT n=15 (All men) | 47-82 yrs | Congestive heart failure according to ICD-9 codes. Ejection fraction ≤0.40.  NYHA* functional stage I-IV |
| ^#^Chang et al. (2004)  USA (Hospital) | Grounded theory  (interviews) | PT n=57 (55 men)  (20 from the intervention group) | Mean=69 | Chronic heart failure  Ejection fraction <0.40.  NYHA* functional stage II-III |
| Evangelista et al. (2001)  USA(OPC) | Qualitative generic  (interviews) | PT n=32 (16 men) | Mean=52.03 SD=12.12 | Heart failure  Ejection fraction (mean ± SD) 26.69 ± 11.46. NYHA* functional stage I-IV |
| Mahoney et al. (2001)  USA (OPC) | Ethnography  (interviews, observation and document review) | PT n= 16  FC n=12 | Not available | Congestive heart failure  Medically diagnosed and treated for CHF |
| Miller et al. (2000)  USA (Hospital and OPC) | Grounded theory  (interviews) | PT n=10^§^ (All women) | 40-78 yrs | Congestive heart failure. Diagnosis of coronary heart disease or congestive heart failure. |
| Rhodes et al. (2002)  USA (Community) | Phenomenology  (interviews) | PT n=5  (All women) | 60-90 yrs | Heart failure  Self-reported NYHA* functional stage II |
| Riegel et al. (2002)  USA (Community) | Qualitative generic  (structured interviews) | PT n=26 (17 men) | 56-91 yrs  Mean=74.4 SD=10.05  Median=76 | Heart failure  Previous hospitalisation due to CHF. Diagnosis confirmed in medical records. Functional status measured using the Specific Activity Scale |
| ^#^Rucker-Whitaker et al. (2006)  USA (Community) | Qualitative generic  (focus groups) | PT n=25 (12 men)  (all from the intervention group) | 39-82 yrs  Mean=55 | Heart failure. HF diagnosed according to ejection fraction^ |
| Scotto et al. (2005)  USA (OPC) | Phenomenology  (interviews) | PT n=14 (9 men) | 42-84 yrs  Mean=63 | Heart failure. Previous hospitalisation due to CHF. |
| Stull et al. (1999)  USA (OPC) | Grounded theory  (interviews and document review) | PT n=21 (17 men) | 29-79 yrs  Mean=61 SD=13.3 | Heart failure. Ejection fraction ≤0.35 and NYHA* functional stage II-IV |
| Westlake et al. (2001)  USA (OPC) | Phenomenology  (interviews) | PT n=87 (60 men) | Mean =56.1  SD=12.9 | Chronic heart failure. NYHA* functional stage  I-IV |
| Zambroski et al. (2003)  USA (Community) | Qualitative generic  (interviews) | PT n=11 (5 men) | 50-81 yrs  Mean=67 | Heart failure. Medically diagnosed and treated for HF |

*NYHA New York Heart Association Criteria. The NYHA classes heart failure into four functional stages according to ejection fraction, with stage IV being the most severe stage

π Outpatient Clinic

^§^ Study included four participants with CHF

^ 82% of participants had an ejection fraction of ≤0.40

^#^ Studies that are conducted as part of the intervention study
